# Macrophage immunometabolism in diabetes-associated atherosclerosis

**DOI:** 10.1097/IN9.0000000000000032

**Published:** 2023-10-16

**Authors:** Bernardo Gindri dos Santos, Leigh Goedeke

**Affiliations:** 1 Department of Medicine (Cardiology), The Cardiovascular Research Institute, Icahn School of Medicine at Mount Sinai, New York, NY, USA; 2 Department of Medicine (Endocrinology), The Diabetes, Obesity and Metabolism Institute, Icahn School of Medicine at Mount Sinai, New York, NY, USA

**Keywords:** macrophage, metabolism, atherosclerosis, diabetes, insulin resistance

## Abstract

Macrophages play fundamental roles in atherosclerotic plaque formation, growth, and regression. These cells are extremely plastic and perform different immune functions depending on the stimuli they receive. Initial in vitro studies have identified specific metabolic pathways that are crucial for the proper function of pro-inflammatory and pro-resolving macrophages. However, the plaque microenvironment, especially in the context of insulin resistance and type 2 diabetes, constantly challenges macrophages with several simultaneous inflammatory and metabolic stimuli, which may explain why atherosclerosis is accelerated in diabetic patients. In this mini review, we discuss how macrophage mitochondrial function and metabolism of carbohydrates, lipids, and amino acids may be affected by this complex plaque microenvironment and how risk factors associated with type 2 diabetes alter the metabolic rewiring of macrophages and disease progression. We also briefly discuss current challenges in assessing macrophage metabolism and identify future tools and possible strategies to alter macrophage metabolism to improve treatment options for diabetes-associated atherosclerosis.

## 1. Introduction

Atherosclerotic cardiovascular disease (ACVD) is a multifactorial chronic disease characterized by the buildup of lipid-rich plaques in medium- and large-size arteries ^[1,2]^. The resulting plaque is the main cause of coronary artery disease and myocardial infarction, accounting for more than 19 million deaths worldwide every year ^[3]^. Several risk factors, such as sex, genetic background, and unhealthy lifestyle choices, are associated with ACVD development ^[2,4]^. However, in the past decades, the growing obesity and metabolic syndrome pandemic have substantially contributed to increasing ACVD incidence ^[5]^. Metabolic syndrome is characterized by several ACVD risk factors, such as dyslipidemia, insulin resistance, hyperglycemia, hypertension, and low-grade inflammation ^[5]^. In addition, metabolic syndrome is strongly associated with the development of type 2 diabetes (T2D), which is well-known to increase atherosclerosis and the incidence of cardiovascular events ^[6]^.

Although diabetic patients do not present significant differences in plaque morphology when compared with nondiabetic ACVD subjects, clinical and experimental data have demonstrated that plaque progression is accelerated while regression is hampered ^[7]^. The underlying mechanism by which ACVD progresses faster in T2D subjects is largely unknown; however, postmortem analyses of plaques have shown increased calcification, macrophage recruitment, and necrotic core area, suggesting that early macrophage inflammatory activation and recruitment to plaques may play an important role in T2D-accelerated atherosclerosis ^[8–10]^. Diabetic and atherosclerotic animal models have also reinforced that macrophages play an important role in plaque progression, with global or macrophage insulin receptor-deficient *Ldlr*^−/−^ mice showing greater macrophage accumulation and necrotic core formation in early and late-stage lesions ^[11]^. More recently, the hyperglycemia-driven myelopoiesis observed in diabetic atherosclerosis regression models has been shown to promote inflammatory monocyte and neutrophil infiltration into the plaque, which strongly hinders plaque regression even under low-density lipoprotein (LDL) cholesterol-lowering conditions ^[12–16]^.

## 2. Macrophage metabolism in atherosclerosis

Macrophages are tissue-resident immune cells that play a fundamental role in ACVD progression ^[17,18]^. Plaque formation is mainly triggered by high circulating LDL cholesterol deposited in the intima of specific vascular regions exposed to high shear stress ^[19]^. During early plaque development, local macrophages uptake the deposited LDL cholesterol and contribute to clearance and tissue maintenance ^[20]^. However, further LDL accumulation exceeds tissue macrophage clearance capacity and induces metabolic dysfunction and oxidative stress, which render them pro-inflammatory and induce the recruitment and differentiation of monocytes from the bloodstream ^[21,22]^. This process ultimately leads to macrophage foam cell formation, contributing to necrotic core formation and plaque burden ^[23,24]^. The rupture of advanced plaques releases the inflammatory and cellular components in the bloodstream and activates blood clot formation and disruption of blood flow and oxygen delivery to tissues ^[19]^.

**Figure 1.**
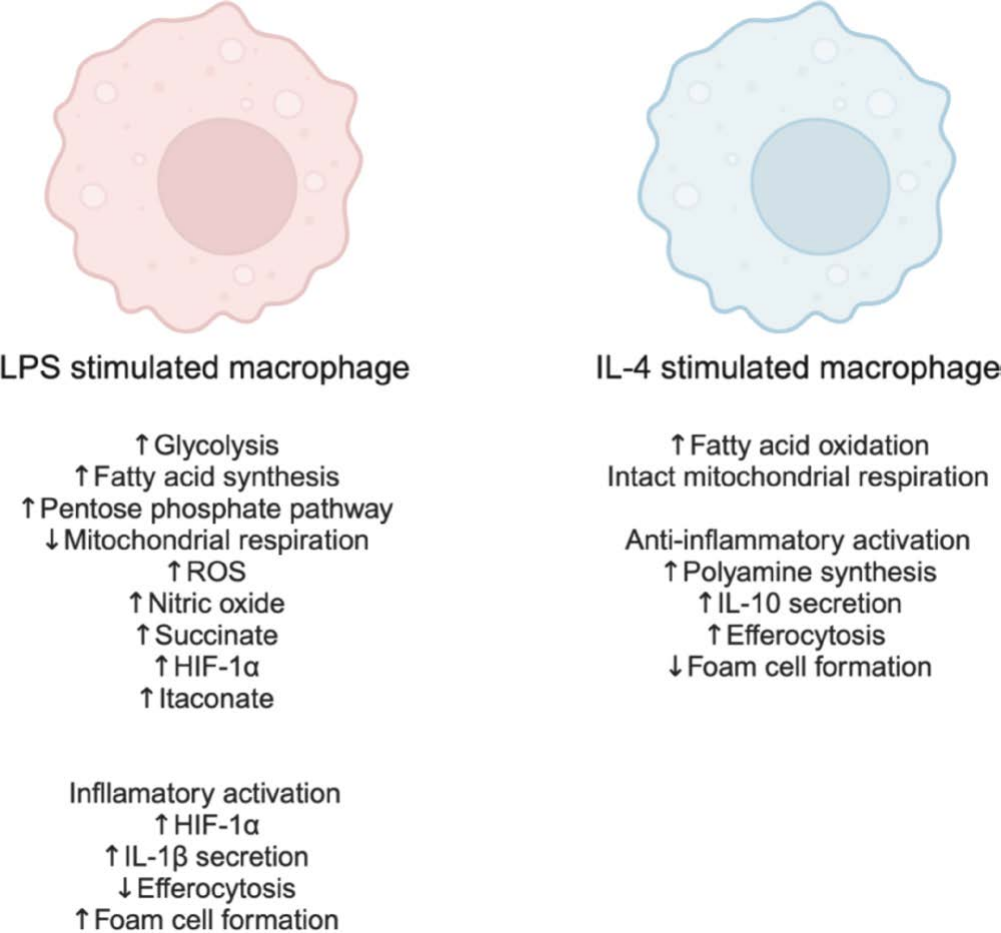
Illustration summarizing the main metabolic and functional differences between pro-inflammatory (LPS stimulated) “M1” and anti-inflammatory (IL-4 stimulated) “M2” macrophages in vitro. IL, interleukin; LPS, lipopolysaccharides; ROS, reactive oxygen species.

Upon exposure to various environmental cues, macrophages can quickly adapt into different functional and metabolic subtypes that play diverse roles in ACVD ^[25]^. The classical view of macrophage polarization is mostly based on in vitro studies that identified two main subtypes: classically activated pro-inflammatory M1 (stimulated by lipopolysaccharides [LPS]) and the alternatively pro-resolving M2 (stimulated by IL-4) subtypes (Figure 1) ^[25,26]^. The M1 polarization state is associated with the secretion of pro-inflammatory cytokines, such as interleukin (IL)-1β and IL-6, reduced efferocytosis capacity, and increased reactive oxygen species (ROS) production ^[27,28]^. This subtype has predominantly been found in rupture-prone large plaques, suggesting that inflammatory-activated macrophages present in the plaque contribute to poor ACVD outcomes ^[23]^. On the other hand, M2 macrophages secrete pro-resolving factors, such as IL-10 and polyamines, and have a higher efferocytosis capacity ^[23,29]^. Not surprisingly, pro-resolving polarized macrophages have been predominantly found in stable plaque regions and regressing plaques, contributing to ACVD resolution ^[23]^.

In addition to altering macrophage phenotypic characteristics, the polarization process also induces profound and specific metabolic rewiring in each macrophage subtype (Figure 1) ^[20]^. One of the main hallmarks in this context is how the oxygen consumption rate (OCR) is reduced in M1 macrophages upon LPS stimulation but remains intact in the M2 polarized subtype ^[25,26,29,30]^. This rearrangement of macrophage metabolism and mitochondrial function completely changes how metabolic substrates (eg, glucose, amino acids, fatty acids, and cholesterol) are used by macrophages to support the specific functions of each subtype, such as pro- or anti-inflammatory cytokine secretion, efferocytosis, and pro-resolving molecule production and release ^[21,25,26]^.

However, the functional and metabolic phenotypes of the classical M1 and M2 macrophage subtypes have mostly been identified in vitro and represent two extremes of a continuum of polarization phenotypes ^[31]^. In the plaque, the microenvironment in which macrophages are localized is different depending on the plaque region and constantly changes during disease progression and regression ^[31]^. Not surprisingly, single-cell RNA sequencing and mass cytometry techniques have shown that exposure to multiple and simultaneous stimuli induces the development of additional macrophage phenotypes such as the proatherogenic Mox and M4 subtypes, and the more anti-inflammatory Mhem and TREM^hi^ macrophages in the plaque ^[32–36]^. In the next sections of this mini review, these aspects of macrophage immunometabolism will be discussed in the context of diabetic atherosclerosis and how the complex plaque environment differentially affects macrophage metabolism and function.

### 2.1 Glucose

Increased glucose uptake and metabolism have been identified as one of the hallmarks of inflammatory-activated macrophages in vitro. Indeed, the understanding of how glucose modulates macrophage function has gained even more attention in the context of atherosclerosis after positron emission tomography techniques demonstrated that glucose uptake was increased in immune cells of human and murine atherosclerotic plaques ^[37–40]^. As briefly mentioned, macrophage polarization strongly alters mitochondrial respiration ^[37,38]^. The reduction in OCR is induced by two breaks in the tricarboxylic acid (TCA) cycle upon macrophage LPS stimulation. The first TCA break increases citrate and *cis*-aconitate levels, which are used as substrates for fatty acid (FA) and itaconate synthesis ^[37,38,41]^. Itaconate is a suppressor of the inflammatory response by reducing inflammatory cytokine release and plays an important role in regulating M1 macrophage activation ^[42,43]^. The second TCA break leads to succinate accumulation, which stimulates ROS production by reverse electron transport, hypoxia-inducible factor 1α (HIF-1α) stabilization, and IL-1β secretion ^[28,37,38]^. HIF-1α is also a strong positive regulator of glycolysis and stimulates the expression of glycolytic and pentose phosphate pathway enzymes and glucose flux through both pathways ^[44,45]^. In contrast, pro-resolving macrophages present an intact OCR and do not rely on glycolysis to polarize into the M2 subtype ^[21,46–48]^.

**Figure 2.**
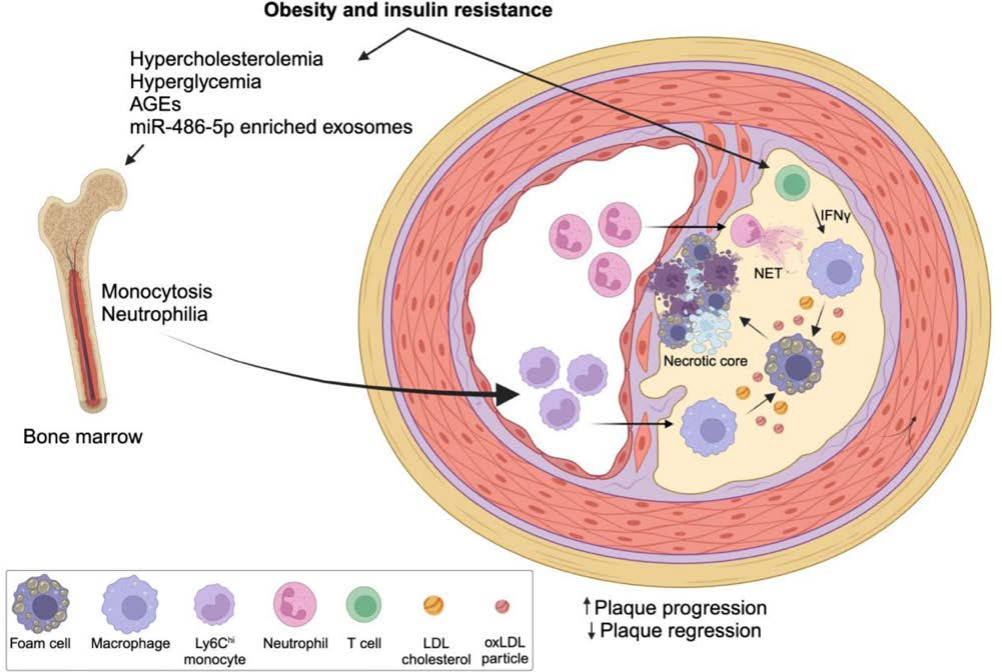
Summary of the main alterations identified in mouse models of diabetic atherosclerosis. Obesity and insulin resistance induce hypercholesterolemia, hyperglycemia, AGEs, and miR-486-5p-enriched exosome secretion which has been shown to increase HSC proliferation and the expansion of inflammatory monocytes and neutrophils, which in turn promote cell infiltration into atherosclerotic lesions, neutrophil extracellular traps (NET) release and macrophage inflammatory activation ^[13,51–53]^. In addition, obesity and insulin resistance may prime T cells to overproduce IFNγ and further stimulate macrophage inflammatory activation and foam cell development in the plaque by increasing the uptake and reducing the efflux of cholesterol ^[54]^. AGEs, advanced glycation end products; HSC, hematopoietic stem cell; IFNγ, interferon-gamma; NET, neutrophil extracellular traps.

One essential part of glucose metabolism is the expression of glucose transporters (GLUTs) to promote glucose influx ^[25]^. Macrophages are known to express GLUT1 and GLUT3, and of those, GLUT1 is strongly upregulated in response to LPS stimulation and is therefore suggested to play a role during diabetic atherosclerosis development ^[49,50]^. Diabetes and hyperglycemia have been shown to modulate hematopoietic stem cell differentiation and promote the progression of atherosclerosis (**Figure 2**) ^[13,51]^. Specifically, hyperglycemia increased myelopoiesis and the expansion of circulating Ly6-C^hi^ monocytes and neutrophils, which promoted cell entry into atherosclerotic lesions ^[13,51]^. In mice, disrupting GLUT1 expression and glucose uptake in hematopoietic cells reduced myelopoiesis, monocyte recruitment to lesions, and plaque necrotic core and size ^[52]^. However, when GLUT1 was overexpressed in *Ldlr*^*−/−*^ mice, it failed to induce inflammation and atherosclerosis, even though myeloid cells presented the same metabolic profile as M1 inflammatory macrophages. Taken together, this suggests that glucose influx by GLUT1 alone is not sufficient to accelerate plaque progression and that the effect of hyperglycemia on increasing myelopoiesis and recruitment of monocytes and neutrophils to the lesion may be driven by additional factors ^[53]^. In addition, a recent study demonstrated that macrophages from diabetic *Ldlr*^*−/−*^ mice presented reduced GLUT1 expression and glycolysis compared with nondiabetic mice, suggesting that increased glucose uptake by tissue-resident macrophages is unlikely to contribute to plaque progression in diabetic atherosclerosis and that factors other than hyperglycemia, such as insulin, may contribute to macrophage metabolic alterations at the tissue level ^[54]^.

Another important consequence of continual or transient hyperglycemia is the generation of advanced glycation end products (AGEs) and the activation of the receptors for advanced glycation end products (RAGE) ^[13,51,55]^. During atherosclerosis, RAGE activation has been implicated as an underlying mechanism inducing monocytosis, neutrophilia, and impaired plaque regression in diabetic mice (Figure 2) ^[13,51]^. At the tissue level, RAGE activation in aortic macrophages increased interferon regulatory factor 7 (Irf7) and similarly reduced atherosclerosis regression in aortas from *Ldlr*^*−/−*^ mice transplanted to WT or *Ager*^*−/−*^ mice, suggesting that RAGE may act as an additional and important mechanism mediating the effects of hyperglycemia on diabetic atherosclerosis progression and regression ^[13,51,55]^.

The glucose source also appears to influence macrophage phenotypes in ACVD ^[56]^. In the plaque microenvironment, macrophages can obtain nutrients by membrane transport, endogenous synthesis, and efferocytosis of apoptotic cells ^[21]^. Increased glycolysis induced by apoptotic cell-derived glucose positively regulates macrophage efferocytosis by increasing solute carrier family 2 member 1 (SLC2A1)/GLUT1 expression ^[56]^. In this case, solute carrier family 16 member 1 (SLC16A) deletion increased necrotic core area and plaque instability, which was associated with reduced efferocytosis, SLC16A downregulation, and lactate release ^[56]^. Extracellular lactate is also an important inducer of IL-10 release in vitro and may stimulate pro-resolving macrophage polarization, tissue repair, and plaque resolution ^[56,57]^. Therefore, intact glycolysis may be necessary to support pro-resolving macrophage function and plaque resolution by promoting continual efferocytosis and lactate release.

### 2.2 Insulin

As discussed in the previous section, the effects of hyperglycemia on macrophage metabolism and function may be associated with additional hyperglycemia-related factors. In the context of diabetes, particularly T2D, hyperglycemia is triggered by defective insulin signaling, which causes insulin resistance and reduced glucose uptake by insulin-sensitive tissues ^[58]^. In the vasculature, insulin regulates macrophages mainly through the phosphoinositide 3-kinase (PI3K)/protein kinase B (AKT) and mammalian target of rapamycin (mTORC) 1 pathway, and its defective signaling has been associated with macrophage endoplasmic reticulum (ER) stress, impaired efferocytosis, and apoptosis ^[11,59–61]^.

To test how macrophage insulin resistance could affect atherosclerosis progression, several groups have generated myeloid-specific insulin receptor (IR) inactivation models in different mouse strains ^[11,62]^. In *ApoE*^*−/−*^ mice, the IR substrate-2 knockout was protective against atherosclerosis, with mice presenting smaller lesions ^[62]^. However, in another study, the transplantation of *IR*^*−/−*^ bone marrow cells to Western-diet-fed *Ldlr*^*−/−*^ mice increased plaque lesion size, apoptotic cells, and necrotic core area, which was related to reduced Akt phosphorylation, increased ER stress, and apoptosis in lesional macrophages ^[58]^. The discrepancies in these two studies may be attributed to several factors, including the different mouse strains used and methods for IR inactivation. Indeed, recent data demonstrated that insulin resistance may prime T cells to overproduce interferon-γ, which consequently affects macrophage metabolism by inducing foam cell formation and worsening atherosclerosis only in obese/insulin-resistant *Ldlr*^*−/−*^ mice ^[63]^.

### 2.3 Amino acids

Amino acid (AA) metabolism is strongly disrupted in diabetes, and the plasma profile of specific AAs has been used to assess diabetes risk and prediction of cardiovascular disease ^[64]^. Specifically, branched-chain and aromatic AA levels have been associated with cardiovascular disease development in diabetic patients by correlating the interquartile scores with anatomical findings such as the intima and media thickness and plaque formation ^[64]^. Although the association may predict clinical outcomes, it is still unclear how plasma AAs may modify macrophage metabolism and contribute to plaque development in diabetes.

Largely explored in vitro, macrophage AA metabolism is profoundly rewired by polarization and directly affects macrophage IL production and the capacity of phagocyte apoptotic cells (ACs). More recently, studies have begun to identify how changes in macrophage AA fate may contribute to nondiabetic atherosclerosis development ^[65,66]^. AC-derived AA catabolism by macrophages has been shown to affect gene expression and plaque resolution by promoting continual efferocytosis in *Ldlr*^*−/−*^ mice ^[65,66]^. Mechanistically, the first uptake of ACs led to high intracellular arginine levels and subsequent putrescine synthesis via arginase-1 and ornithine decarboxylase enzymes, which were upregulated in pro-resolving macrophages ^[65]^. Putrescine then acted by enhancing Dbl expression and Rac1 activity, promoting cytoskeleton rearrangement, a second uptake of ACs, and continual efferocytosis ^[65]^.

In addition to directly stimulating macrophage functions such as efferocytosis, AA metabolism may also induce epigenetic modifications that result in the secretion of pro-resolving factors ^[66]^. Such a mechanism has been identified with AC-derived methionine, in which metabolism into *S*-adenosylmethionine repressed phosphatase Dual-specificity phosphatase 4 (DUSP4) gene expression via DNA methylation by DNA-methyltransferase 3A (DNMT3A). DUSP4 repression allows continual activation of the extracellular signal-regulated kinase 1/2, which induces prostaglandin E_2_ and tumor growth factor-β1 secretion, promoting efferocytosis, tissue repair, and reduction in plaque size ^[66]^.

Glutamine metabolism is also strongly affected by macrophage polarization and has been implicated in supporting both inflammatory and pro-resolving phenotypes in atherosclerosis ^[67,68]^. The first step of glutaminolysis is the conversion of glutamine to glutamate by glutaminase (GLS) ^[69]^. Next, glutamate can undergo oxidative deamination catalyzed by glutamate dehydrogenase (GLUD) or transamination catalyzed by aminotransferases, such as aspartate aminotransferase (GOT), producing α-ketoglutarate, which can fuel the TCA cycle ^[69]^. LPS-stimulated macrophages exposed to damage-associated molecules such as oxidized phospholipids displayed a hyperinflammatory and oxidative phenotype and were associated with worsened atherosclerosis in *ApoE*^*−/−*^ mice fed a hypercholesterolemic diet ^[68]^. This phenotype was dependent on glutamine catabolism by GLS and GLUD, fueling the TCA cycle and promoting citrate accumulation, which was ultimately exported to the cytoplasm and converted to oxaloacetate (OAA) ^[68]^. Cytoplasmic OAA activated HIF-1α and IL-1β, promoting hyperinflammation even upon electron transport chain (ETS) inhibition ^[68]^.

In contrast, glutamine catabolism by GLS1 has also been implicated in promoting macrophage continual efferocytosis, oxidative phosphorylation (OXPHOS) maintenance, antioxidant production, and plaque reduction ^[67]^. Specific myeloid cell GLS1 deletion increased plaque area and necrotic core by reducing local macrophage efferocytosis ^[67]^. Interestingly, in vitro data demonstrated that GLS1-derived glutamate was fueling the TCA cycle and promoting reduced glutathione (GSH) regeneration through the malate-aspartate shuttle and GOT activity in pro-resolving macrophages ^[67]^. GSH is the most important nonenzymatic endogenous antioxidant, and its upregulation was associated with reduced oxidative stress and plaque resolution ^[67]^. In addition, this data also provides compelling evidence that even subtle metabolic rewiring, as in the case of glutamate fate, allows macrophages to support completely different functions while simultaneously maintaining high OCR, a metabolic characteristic previously attributed to pro-resolving macrophages ^[25]^.

### 2.4 Fatty acids

Macrophage polarization through LPS or IL-4 stimulation is known to differentially affect FA metabolism in vitro ^[23,70]^. LPS-activated macrophages generally present increased FA synthesis while pro-resolving macrophages depend on FA oxidation (FAO), though such a notion has been challenged by some studies demonstrating that long-chain FAO is dispensable for M2 macrophage polarization ^[71,72]^. Macrophages can obtain lipids using multiple mechanisms, such as the FA transport proteins, lipoprotein lipase (LPL), and CD36 ^[73–75]^. As mentioned above, atherosclerotic plaques have high lipoprotein and oxidized lipid contents, which strongly induce macrophage inflammatory activation and lipid uptake by CD36 and LPL activities ^[23,70,76,77]^. In diabetic patients, high serum CD36 levels have long been associated with insulin resistance and hyperglycemia, and recently, its expression in circulating monocytes has been strongly correlated with poor glycemic control and coronary artery disease risk in diabetic subjects ^[78,79]^. In mice, specific bone marrow cell deletion of CD36 and LPL decreased plaque size and foam cell number (a common characteristic of advanced plaques) by reducing macrophage FA accumulation, demonstrating that targeting lipid uptake may be a therapeutic strategy to prevent macrophage dysfunction ^[23,70,76,77]^.

Following uptake, FAs may bind to FA-binding proteins (FABP), which control FA intracellular distribution ^[80]^. To date, nine different FABPs have been identified, but their role in regulating macrophage metabolism and phenotype in atherosclerosis is still largely unknown ^[81]^. A few studies, however, have shown that bone marrow cell deletion of FABP4 and FABP5 reduced plaque size and inflammatory markers in the aorta of *ApoE*^−/−^ mice fed a high-fat cholesterol diet ^[82,83]^. Interestingly, reducing FABP-associated FAs enhanced peroxisome proliferator-activated receptor γ, CD36, and adenosine triphosphate (ATP) binding cassette subfamily A member 1 (ABCA1) expression in macrophages, increasing FA and cholesterol influx and efflux simultaneously ^[82,83]^. FABP4 deletion also downregulated macrophage-inducible nitric oxide synthase expression, which may alternatively explain the reduced inflammatory phenotype because nitric oxide-induced ETS inhibition has been shown to be an important step in IL-1β production in LPS-stimulated macrophages ^[83,84]^.

Saturated and monounsaturated FA synthesis catalyzed by FA synthase (FAS) is an important step in controlling FA metabolism in macrophages ^[85–87]^. Once synthesized, FAs may deposit in lipid droplets, incorporate into membrane and cell compartments, or be used as substrates in the synthesis of FA-based molecules ^[85–87]^. Macrophage-specific FAS deletion improved atherosclerosis in mice by reducing plaque size by 30% and foam cell number ^[88]^. Mechanistically, macrophages presented enhanced liver X receptor (LXR) expression, which induced ABCA1 upregulation and CD36 downregulation, promoting cholesterol efflux and preventing LDL uptake ^[88]^. Another important enzyme involved in FA synthesis is ATP-citrate lyase (ACLY), which converts mitochondria-derived citrate into cytosolic acetyl-CoA and supports macrophage inflammatory activation by regulating histone acetylation and prostaglandin synthesis ^[89–92]^. Myeloid-specific *Acly* knockout reduces atherosclerosis in *Ldlr*^*−/−*^ mice by increasing macrophage efferocytosis capacity ^[89]^.

On the other hand, FAs may also be redirected to the TCA cycle to support mitochondrial respiration and ATP production ^[93]^. One of the main steps during FAO is the activation of acyl-CoA by acyl-CoA synthetase (ACSL), which produces acyl-carnitine and allows transport into the mitochondria ^[94]^. ACSL1 has been found to be upregulated in inflammatory-activated macrophages in vitro, and its deletion reduced FA utilization without affecting mitochondrial respiration ^[93]^. Interestingly, myeloid cell-specific *Acls1* deletion reduced atherosclerosis development and inflammatory activation in diabetic *Ldlr*^*−/−*^ mice but not in nondiabetic mice, suggesting a role of myeloid cell FA metabolism in accelerated T2D atherosclerosis, though the exact mechanism remains unknown ^[94]^.

### 2.5 Cholesterol metabolism

The regulation of cholesterol metabolism has been shown to be altered during atherosclerosis ^[95–97]^. Macrophage cholesterol uptake is mediated by different mechanisms, but in the setting of atherosclerosis, most cholesterol import is mediated by CD36 and efferocytosis of AC ^[95–97]^. The higher uptake of oxidized LDL (oxLDL) particles and cholesterol crystals by CD36 has been shown to enhance inflammasome activation and IL-1β synthesis by macrophages in vitro ^[95–97]^. Therefore, the high lipid content in plaques, especially oxLDL particles, may induce inflammatory activation of local macrophages, which may also explain why CD36 downregulation reduces plaque size in vivo, especially when accompanied by increased cholesterol efflux ^[74,88]^.

Cholesterol metabolism is coordinated by a series of transcription factors and membrane transporters that adjust cholesterol biosynthesis, import, and export ^[98]^. LXR is one of the main transcription factors controlling cholesterol homeostasis by inducing ABCA1 expression and cholesterol export ^[98]^. LXR, in turn, is regulated by desmosterol, an intermediate metabolite in cholesterol biosynthesis ^[99,100]^. Desmosterol depletion in myeloid cells is strongly correlated with abnormal cholesterol and lipid metabolism, macrophage inflammatory activation, and foam cell formation, which promotes atherosclerosis in mice ^[99,100]^. In addition, AC engulfment by efferocytosis increases intracellular cholesterol and oxysterol content, leading to the upregulation of LXR and ABCA1, which was mediated by the efferocytosis of receptors, lipoprotein receptor-related protein 1, and brain-specific angiogenesis inhibitor 1 ^[101–103]^. Together, these studies demonstrate that impaired cholesterol efflux affects macrophage AC phagocytic capacity, contributing to foam cell formation and the progression of atherosclerosis. Targeting cholesterol metabolism in aortic macrophages by modulating desmosterol and/or LXR activation may offer important therapeutic strategies to hinder plaque progression.

### 2.6 Mitochondrial function

Mitochondria health and ROS production are additional important factors contributing to cholesterol homeostasis and macrophage function in atherosclerosis ^[104]^. The nicotinamide adenine dinucleotide phosphate (NADPH)-oxidase enzymes and mitochondrial ETS are the main ROS sources in the cell ^[105]^. In basal conditions, ROS acts as signaling molecules by oxidizing cysteine residues in target proteins such as NRF2 and the nuclear factor kappa-light-chain-enhancer of activated B cells (NFκB) ^[106]^. However, when ROS production exceeds the cell’s detoxification capacity, it can lead to oxidative stress which damages cell structures and consequently activates cell death and inflammation ^[107]^. Oxidative stress and mitochondrial dysfunction are common hallmarks of atherosclerotic plaques, particularly in the hypoxic regions close to the necrotic core ^[108–110]^. This may happen, as mentioned above, by ROS overproduction or by depletion of antioxidant defenses, such as superoxide dismutase 1, in the aorta of diabetic mice ^[111]^. High ROS generation leads to membrane damage and mitochondrial DNA (mtDNA) release into the cytosol, which is closely related to nucleotide-binding domain, leucine-rich-containing family, pyrin-domain-containing-3 (NLRP3) inflammasome activation in macrophages ^[110,112–114]^. High levels of mtDNA have also been found in the peripheral blood of diabetic patients with atherosclerosis when compared with diabetic or ACVD patients only ^[115]^.

In addition to damaging mitochondrial membranes, ROS can also dysregulate mitochondrial biogenesis and dynamics ^[116]^. Both mitochondrial biogenesis and dynamics are fundamental processes that mediate cellular metabolic adaptations, and their dysregulation has been associated with atherosclerosis and plaque progression ^[116]^. Mitochondrial biogenesis is governed by the transcription factor peroxisome proliferator-activated receptor-gamma coactivator 1α (PGC1α), which simultaneously induces nuclear and mitochondrial gene expression and the synthesis and integration of new mitochondria into the cell mitochondria network ^[117]^. PGC1α expression is downregulated in plaques of hypercholesterolemic mice, and its repression in macrophages impaired mitochondrial biogenesis, ATP production, and cholesterol efflux through ABCA1, suggesting that intact mitochondrial function is necessary for cholesterol homeostasis and can prevent foam cell formation ^[118,119]^.

Once new mitochondria are integrated into the cell’s mitochondrial network, they start to participate in mitochondrial dynamics ^[120]^. This process consists of two distinct mechanisms: mitochondrial fission and mitochondrial fusion ^[121]^. Mitochondrial fission is mainly mediated by the recruitment of dynamin-related protein 1 (DRP1) around the target mitochondrion and its cleavage into two smaller mitochondria ^[122]^. In contrast, mitochondrial fusion is mediated by mitofusin (MFN) 1 and 2, and optic atrophy 1 (OPA1) proteins, fusing two small mitochondria into one ^[122]^. DRP1-mediated mitochondrial fission was upregulated in macrophages during continual efferocytosis, and both DRP1 deletion and mitochondrial fission inhibition impaired AC continual clearance and increased plaque necrotic core and AC content ^[123]^. The authors established a mitochondrial-dependent calcium mechanism regulating phagosome sealing and AC degradation; however, the metabolic consequences of a fragmented mitochondrial network and how it can reshape macrophage metabolism during efferocytosis remain to be determined ^[123]^.

In addition, the post-transcriptional regulation of genes controlling mitochondrial physiology by microRNA-33 (miR-33) can also influence cholesterol metabolism and plaque formation ^[118,124]^. In the plaque, miR-33 is overexpressed, which correlates with reduced mitochondrial function and ATP production ^[118]^. Suppression of miR-33 levels in atherosclerotic mice restored mitochondrial health by promoting PGC1α and NRF1 upregulation, increasing cholesterol efflux mediated by ABCA1 and ATP production ^[118,124]^. In diabetic mice, miR-33 suppression strongly reduced plaque size in an atherosclerosis regression model ^[125]^. Such an effect was attributed to reduced macrophage inflammatory activation and traffic in the aorta, in addition to lower bone marrow progenitor cell differentiation and total monocyte population expansion when compared with diabetic control mice ^[125]^. Therefore, preventing or reversing macrophage mitochondrial and cholesterol metabolism dysfunction may reduce plaque burden and atherosclerosis in vivo, demonstrating again that the mitochondria represent an important nexus for controlling macrophage activation and metabolism ^[109,118,124]^.

### 2.7 Extracellular vesicles

Exosome secretion is an essential mechanism by which cells can exchange nutrients and signaling molecules ^[126]^. These nanoscale-sized extracellular vesicles (EVs) carry a complex combination of proteins, lipids, carbohydrates, and genetic material, which promote long-distance intercellular communication and regulation of recipient cell function ^[127]^. Recent evidence also suggests that exosome cargo secreted by distinct cell types can modulate macrophage polarization between inflammatory and anti-inflammatory profiles, thus representing a new target to treat inflammation associated with metabolic diseases ^[128,129]^.

Particularly in the context of diabetic atherosclerosis, systemic metabolic alterations have been shown to aggravate plaque progression by altering macrophage-derived exosome content and lesion inflammation ^[130,131]^. Specifically, exposure of cultured macrophages to oxLDL increases exosome miR-146a content and potentiates atherosclerosis in *ApoE*^*−/−*^ mice by promoting neutrophil extracellular traps ^[131]^. Similarly, exosomes produced by bone marrow-derived macrophages (BMDMs) under hyperglycemic conditions exhibit high miR-486-5p levels, and when infused in high-fat cholesterol diet-fed *ApoE*^*−/−*^ mice, increase myelopoiesis and atherosclerosis progression (Figure 2) ^[130]^. Further analysis revealed that plasma EVs from diabetic patients with advanced lesions also have enriched miR-486-5p content ^[130]^. When co-cultured with naïve macrophages, diabetic EVs were able to reduce OXPHOS gene expression, suggesting a possible feedforward mechanism by which metabolically stressed macrophages can communicate with target cells ^[130]^.

Macrophage, mesenchymal stem cell (MSC), and dendritic cell-derived exosomes can also induce macrophage anti-inflammatory polarization and have been shown to improve atherosclerosis ^[132,133]^. Continual stimulation of BMDMs with IL-4 was shown to increase exosome miR-146b, miR-99a, and miR-378a, which reduced inflammation by NF-κB and tumor necrosis factor-α (TNF-α) in target macrophages ^[132]^. IL-4-stimulated BMDM exosome infusion to *ApoE*^*−/−*^ mice reduced aortic root lesions by controlling hematopoiesis and reducing myeloid cell recruitment to lesions ^[132]^. Exosomes derived from IL-4-treated Tohoku Hospital Pediatrics-1 (THP-1) macrophages also have reduced miR-33 expression, suggesting a role for these exosomes in controlling cholesterol efflux and foam cell formation ^[132,134]^. Other important cells regulating the immune response, such as MSCs and dendritic cells, have been shown to secrete exosomes capable of inducing an M2-like phenotype in target macrophages by increasing miR-let7 and miR-203-3p transfer and reducing plaque size and macrophage infiltration in high-fat fed *ApoE*^*−/−*^ mice ^[135,136]^. Together these studies demonstrate that exosomes secreted by macrophages and other cell types may influence plaque macrophage inflammatory polarization and provide another important therapeutic alternative to manage diabetic atherosclerosis.

## 3. Conclusions and future perspectives

Understanding macrophage metabolism has proven to be essential for uncovering novel mechanisms by which the tissue microenvironment modulates macrophage immune function. In vitro data have provided important directions of how macrophage metabolism is rewired upon different types of pro- or anti-inflammatory stimulation (Figure 1), but the in vitro phenotype does not completely represent how metabolism coordinates macrophage responses in the complex tissue microenvironment. In the case of atherosclerosis, it is important to consider that plaque composition changes in a timely manner as the disease progresses and constantly challenges tissue-resident macrophages with different stimuli such as ROS, oxidized lipids, hypoxia, apoptotic cells, exosome cargo, and high glucose and insulin, in the case of T2D (Figure 2). Therefore, understanding how metabolic flux is modulated in plaque-resident macrophages during disease progression will be fundamental to uncovering new therapeutic opportunities. However, several technical limitations will need to be overcome to obtain more robust and reproducible data. One of the main challenges in profiling cell populations in the aorta is the low yield of viable cells, especially after intensive digestion protocols that are used to obtain single-cell suspensions, which besides reducing cell viability, may also affect cell membrane protein composition and the level of short-lived metabolites. The use of techniques that allow for the identification and distribution of metabolites in the tissue, such as spatial metabolomics, may improve our understanding of specific macrophage populations and help reveal new subtypes that may emerge as the plaque progresses ^[137,138]^. The development of new techniques that identify and integrate cellular gene expression patterns, protein and other macromolecule modification, and metabolite flux at the subcellular level in a time-sensitive and spatial manner will also aid in this endeavor and potentially reveal new therapeutic targets at different time points during plaque development.

In addition, as discussed throughout the review, the source and mechanisms by which macrophages obtain nutrients in the plaque will also determine immune response and efferocytosis capacity, which ultimately contribute to plaque repair. Another important consideration is how myeloid cell metabolic memory influences atherosclerosis progression and regression, especially in T2D. The concept of trained innate immunity has continuously demonstrated that the metabolic syndrome and T2D milieu (hyperlipidemia, hypercholesterolemia, hyperglycemia, and insulin resistance) may induce a pro-inflammatory phenotype in monocytes in the circulation and promote recruitment to plaque lesions ^[52,139]^.

Mitochondria are fundamental in controlling cellular metabolic adaptations and macrophage response. Therefore, a better understanding of how the dysmetabolic and hyperinflammatory state in the plaque affects macrophage mitochondrial function may reveal novel therapeutic strategies. Specifically, mitochondria-targeted compounds (such as mitoquinol [MitoQ], mitochondrial uncoupling agents, and antioxidant compounds) or exosome-derived miRNAs may hold the potential to prevent/revert organelle dysfunction and modulate core signaling pathways mediating mitochondrial and nuclear communication, such as AMP-activated protein kinase (AMPK), AKT-mTOR, and sirtuins ^[133,140]^. In addition, half of cellular ROS is produced by mitochondria, but the mechanisms of how ROS-mediated signaling controls macrophage function during atherosclerosis are mostly unknown. Although short-lived, superoxide radicals and hydrogen peroxide have a strong signaling capacity by specifically oxidizing cysteine residues in target proteins and modulating signaling pathways, enzymes, and endogenous antioxidants that control cellular metabolic regulation and stress responses.

ACVD still remains the leading cause of death in diabetic patients, and targeting macrophage immunometabolism has emerged as a potential and promising therapeutic option, particularly after clinical findings have demonstrated that controlling inflammation may reduce atherosclerosis ^[141]^. Diabetic ACVD is complex with multiple factors, including hyperglycemia and insulin resistance, contributing to accelerated plaque development and impaired regression. In this context, traditional medications for diabetes, such as metformin and SGLT2 inhibitor may help control hyperglycemia and insulin resistance and reduce ACVD events ^[142,143]^. In addition, available data suggest that both compounds affect macrophage inflammatory activation and metabolism ^[144–146]^. Thus, as technical advancements improve our understanding of macrophage and myeloid cell metabolism in ACVD, new therapeutic opportunities will be identified and allow for the development of more targeted approaches to reduce tissue macrophage inflammatory activation by specifically modulating its metabolism and treating ACVD at any stage, particularly in high-risk T2D patients.

## Author contributions

BGdS and LG designed the review article. BGdS wrote and edited the review article and made figures. LG edited the article and had primary responsibility for final content. All authors read and approved the final version of the article.

## Conflicts of interest

The authors declare that there are no conflicts of interest.

## Funding

This work was supported by a grant from the National Institutes of Health (R00 HL150234 to LG).

## Acknowledgments

Figures were created using BioRender.com.
